# Microtubule associated protein WAVE DAMPENED2-LIKE (WDL) controls microtubule bundling and the stability of the site of tip-growth in *Marchantia polymorpha* rhizoids

**DOI:** 10.1371/journal.pgen.1009533

**Published:** 2021-06-04

**Authors:** Clement Champion, Jasper Lamers, Victor Arnold Shivas Jones, Giulia Morieri, Suvi Honkanen, Liam Dolan

**Affiliations:** Department of Plant Sciences, University of Oxford, Oxford, United Kingdom; The University of North Carolina at Chapel Hill, UNITED STATES

## Abstract

Tip-growth is a mode of polarized cell expansion where incorporation of new membrane and wall is stably restricted to a single, small domain of the cell surface resulting in the formation of a tubular projection that extends away from the body of the cell. The organization of the microtubule cytoskeleton is conserved among tip-growing cells of land plants: bundles of microtubules run longitudinally along the non-growing shank and a network of fine microtubules grow into the apical dome where growth occurs. Together, these microtubule networks control the stable positioning of the growth site at the cell surface. This conserved dynamic organization is required for the spatial stability of tip-growth, as demonstrated by the formation of sinuous tip-growing cells upon treatment with microtubule-stabilizing or microtubule-destabilizing drugs. Microtubule associated proteins (MAPs) that either stabilize or destabilize microtubule networks are required for the maintenance of stable tip-growth in root hairs of flowering plants. NIMA RELATED KINASE (NEK) is a MAP that destabilizes microtubule growing ends in the apical dome of tip-growing rhizoid cells in the liverwort *Marchantia polymorpha*. We hypothesized that both microtubule stabilizing and destabilizing MAPs are required for the maintenance of the stable tip-growth in liverworts. To identify genes encoding microtubule-stabilizing and microtubule-destabilizing activities we generated 120,000 UV-B mutagenized and 336,000 T-DNA transformed *Marchantia polymorpha* plants and screened for defective rhizoid phenotypes. We identified 119 mutants and retained 30 mutants in which the sinuous rhizoid phenotype was inherited. The 30 mutants were classified into at least 4 linkage groups. Characterisation of two of the linkage groups showed that MAP genes–*WAVE DAMPENED2-LIKE* (*WDL*) and *NIMA-RELATED KINASE* (*NEK*)–are required to stabilize the site of tip growth in elongating rhizoids. Furthermore, we show that MpWDL is required for the formation of a bundled array of parallel and longitudinally orientated microtubules in the non-growing shank of rhizoids where MpWDL-YFP localizes to microtubule bundles. We propose a model where the opposite functions of MpWDL and MpNEK on microtubule bundling are spatially separated and promote tip-growth spatial stability.

## Author summary

Plant cells control where they grow by adding membrane and cell wall material to a defined area of their surface. In particular, filamentous rooting cells develop the cellular projections essential to their function by restricting cell expansion to a stable domain of their surface. The spatial stability of this mechanism known as tip-growth defines the final shape of the cellular projections–straight projections form from stable tip-growth, while wavy or bifurcating projections form from unstable tip-growth. Microtubules are known to regulate tip-growth stability. Both microtubule stabilisation and destabilisation leads to unstable tip-growth. We have discovered two proteins that associate with microtubules, control their stability and are required for stabilizing tip-growth in the common liverwort. The first protein is known to destabilize microtubules in the tip of filamentous rooting cells of the common liverwort, and we found the second protein to stabilize, or bundle, microtubules in their shank. This is important because it is the first protein found to stabilize microtubules in the common liverwort and because it is the first time a protein stabilizing microtubules in rooting cells of plants is shown to localize separately from proteins that destabilizes microtubules. We propose that tip-growth stability requires the opposite functions of these two microtubule associated protein to be spatially separated.

## Introduction

Filamentous cells, such as root hairs of vascular plant sporophytes and rhizoids on vascular and non-vascular plant gametophytes, form at the interface between plants and soil. They carry out rooting functions, such as anchorage, water and nutrient uptake, and interact with microorganisms [1]. Their tubular shape is key to this function because anchorage is defective in *Marchantia polymorpha* mutants with defective rhizoid morphology (S1 Fig) and in *Arabidopsis thaliana* mutants with defective root hairs [2]. Filamentous rooting cells elongate by tip-growth, a mechanism where growth is stably restricted to a small domain of the cell surface from which the tubular projection grows. Root hairs and rhizoids form as straight cylinders when growing in the air. However, when growing through soil substrates their growth direction continually changes as the tip manoeuvres around objects in the soil.

Tip growth involves the delivery to the apical dome of secretory vesicles containing cell wall material in land plants [3], including liverworts (S3A Fig). The stability of the position of the growing tip is controlled by external factors, such as soil particles, and internal factors such as microtubules [4–6]. A common feature of the microtubule array in tip-growing cells of all land plants is its organization in the shank of the cell, proximal to apical dome. In the non-growing shank, bundled cortical microtubules and endoplasmic microtubules are oriented longitudinally and grow towards the tip [7–13]. The conservation of this organization of microtubules suggests that parallel, longitudinal microtubules growing from a basal position in the non-growing shank to the apical dome is required for tip-growth.

A function of the microtubule cytoskeleton in stabilizing tip-growth has been inferred from observations of plant tip-growing cells treated with drugs that inhibit microtubule assembly and microtubule organization. Tip-growing cells exposed to drugs that either stabilize or destabilize microtubules display wavy tubular morphologies, suggesting that the growth region is unstable and shifts laterally from an apical position to a subapical position [6,11]. Branching root hairs are also observed following the application of microtubule stabilizing drugs, suggesting that dynamic microtubules are required to maintain a single growth point. These observations suggest that the regulation of microtubule dynamics is required to stabilize the position of the growing region in tip-growing cells. Consistent with this hypothesis, loss of function mutations in genes coding for microtubule associated proteins (MAPs) that promote microtubule growth or destabilize microtubules can lead to loss of tip-growth spatial stability. In *A*. *thaliana*, MICROTUBULE ORGANIZATION1 (MOR1) promotes microtubule growth and bundling, and the temperature-sensitive mutant *mor1-1* forms wavy root hairs at the restrictive temperature [14]. By contrast, ARMADILLO-REPEAT KINESIN1 (ARK1) destabilizes microtubules by promoting the transition of microtubules from the polymerizing to the depolymerizing state, and root hairs are wavy in the *ark1* loss of function mutant [15]. In the liverwort *Marchantia polymorpha*, the microtubule destabilizing MAP NIMA-RELATED PROTEIN (MpNEK) is required to maintain a stable position of the growing region in rhizoids [16]. However, no microtubule stabilizing MAP has been reported to function in stabilizing tip-growth in the liverwort model to date.

We hypothesized that microtubule stabilizing MAPs may also be required in tip-growing cells of liverworts to stabilize the position of the growth point. To identify microtubule stabilizing MAPs that stabilize tip-growth in liverworts, we screened T-DNA- and UV-mutagenized *Marchantia polymorpha* populations for mutants with wavy rhizoids that are morphologically similar to oryzalin-treated or Taxol-treated rhizoids. We isolated two *nek* mutants, demonstrating that mutants with defective microtubule organization were identified in the screen [16]. Moreover, we identified four mutant lines with T-DNA insertions in the gene encoding the MAP, *WAVE DAMPENED2 LIKE* (*WDL*). We show that MpWDL localizes preferentially to microtubules in the non-growing shank, where it promotes the bundling and parallel organization of longitudinal microtubules. Together, these results indicate that MAPs with opposite effects on microtubule bundling–MpNEK depolymerizes while MpWDL bundles–are required to regulate the organization of the microtubule array that stabilizes the site of tip-growth in liverwort rhizoid.

## Results

### Mutant screen identified 30 mutants with putative defects in microtubule dynamics

**Fig 1:**
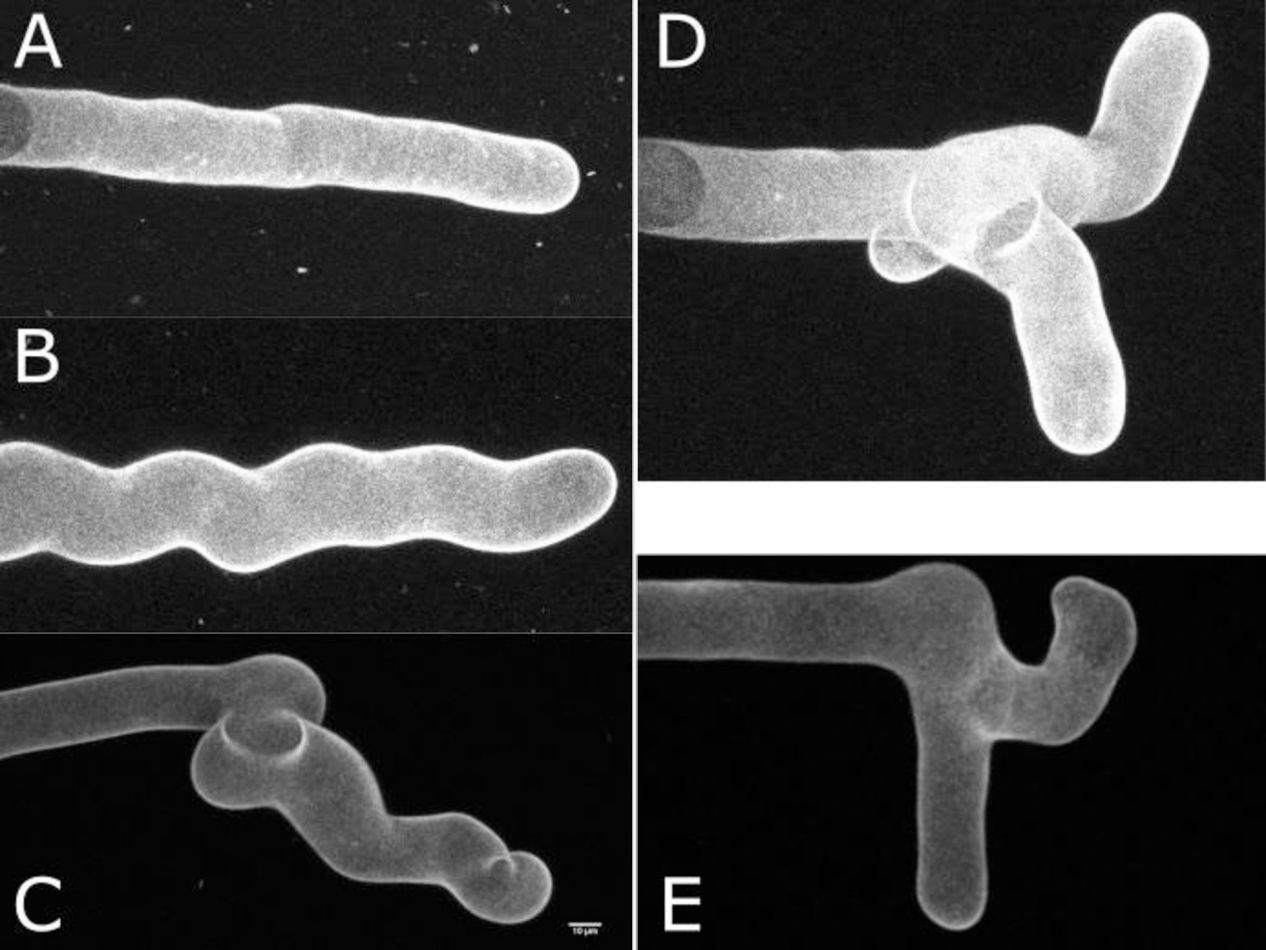
Rhizoid cells treated with the microtubule-destabilizing drug oryzalin or the microtubule-stabilizing drug Taxol form wavy or branched tubular projections. Z-maximum projection of propidium iodide-stained mature rhizoids grown on 0.1% DMSO (**A**), 0.1 μM oryzalin (**B**) or 3.3 μM Taxol (**C**), 0.75 μM oryzalin (**D**) and 5 μM Taxol (**E**).

To identify proteins that are active in microtubule-mediated stabilization of the apex during tip-growth, we screened mutagenized *Marchantia polymorpha* plants for mutants with defective rhizoids. To define the expected rhizoid phenotypes of mutants with defective microtubule organization, we first defined the morphology of wild type rhizoids with impaired microtubule dynamics caused by oryzalin-treatment, which inhibits microtubule polymerisation at the plus-end, and Taxol-treatment, which stabilizes microtubules by inhibiting depolymerisation at both plus and minus ends. Rhizoids were grown in the presence of the microtubule-depolymerizing drug oryzalin (Fig 1) and observed with a confocal microscope. At lower oryzalin concentrations (0.1 μM– 0.5 μM), rhizoids developed a wavy phenotype compared to rhizoids grown in control conditions which were straight. At higher oryzalin concentrations rhizoid sinuosity increased and some rhizoids branched. Similarly, growth of rhizoids in the presence of 3.3 μM Taxol resulted in a wavy rhizoid morphology and branched rhizoids. Taken together, these data indicate that that microtubules are not required for rhizoid growth per se. However, it suggests that a dynamic network of microtubules is required to stabilize the position of the apical domain of the tip growing rhizoid.

To identify genes that control the stability of the growing apex in rhizoids we carried out forward genetic screens for mutants that have rhizoids resembling oryzalin- and Taxol-treated rhizoids. 120,000 UV-B mutagenized plants and 336,000 T-DNA transformed lines were generated and screened for wavy rhizoid phenotypes. We initially selected 97 independent mutants with wavy rhizoids on the mature gametophyte from the 120,000 UV-B mutagenized plants in the initial screens. We also selected 22 wavy rhizoid mutants from the 336,000 T-DNA transformed lines [17]. Of these 119 mutants, 30 (22 UV-B-induced wavy mutants and 8 T-DNA-induced wavy mutants) were retained for further analysis. The remaining 89 mutants were not characterized further in this study, either because their mutant phenotype could not be observed in subsequent vegetative generations or because their growth was too severely stunted.

To quantify the defective phenotypes of rhizoids in these mutant lines, rhizoids that formed on the upper side of gemmae, which are vegetative propagules genetically identical to the plant from which they form, were imaged two days after plating. Two parameters were measured: rhizoid diameter (thickness) and rhizoid sinuosity (waviness) (Table 1). An Anova test indicated that wild type and mutants could be discriminated by rhizoid sinuosity and rhizoid diameter (p_kruskal-wallis_ < 2.10^−6^). Therefore, these two phenotypic parameters were used to categorize the mutant lines.

**Table 1:** Rhizoid phenotypes of T-DNA and UV mutant lines fall in two categories. The mean values of rhizoid sinuosity and rhizoid diameter were measured in 10 mature rhizoids per genotype and are given +/- SD. Single or double stars indicate a Benjamini-Hochberg adjusted p-value from non-parametric Dunn test lower than 0.05 and 0.01 respectively. Phenotypes significantly different from wild type were highlighted to help visualize the independence of the large rhizoid and wavy rhizoid phenotypes.

Mutagenesis	Lines	Rhizoid sinuosity (%)	Rhizoid diameter (μm)	Gaping air pores	Phenotypic group
none	Tak-1	100.5 +/-0.2	20.8 +/-2.1		Wild type
	Tak-2	100.9 +/- 1.2	19.5 +/-2.4		Wild type
T-DNA insertion	ST45-1	110.1 +/-5.6**	19.1 +/- 2.0		2
	CR2	112.6 +/-14.9**	19.5 +/-2.1		2
	CR1	105.8 +/-7.2**	18.9 +/-1.9		2
	ST33-5	103.5 +/-8.4	18.9 +/-1.4		
	ST33-1	103.2 +/-4.7	32.7 +/-3.7**	yes	1
	SH21	105.0 +/-7.1**	15.7 +/-1.6*		
	ST47-6	114.8 +/-44.4*	17.7 +/-2.5		
	VJ72	104.6 +/- 8.0 **	18.0 +/- 2.3		2
UV-B irradiation	UV3.4	103.6 +/-2.5**	23.2 +/-3.0		2
	UV3.8	136.8 +/-52.7**	22.2 +/-2.5		2
	UV3.13	114.7 +/-9.3**	21.8 +/-4.1		2
	UV4.31	105.4 +/-6.9**	27.6 +/-2.3**	yes	1
	UV4.32	101.5 +/-1.2	25.7 +/-1.5**	yes	1
	UV4.34	105.8 +/-11.4*	29.4 +/-2.4**	yes	1
	UV5.5	108.3 +/-7.2**	19.8 +/-1.4		2
	UV5.8	103.4 +/-4.3	23.1 +/-2.6		
	UV5.21	NA	NA		
	UV5.28	100.6 +/-0.4	27.4 +/-4.9**	yes	1
	UV5.30	114.8 +/-16.9**	18.88 +/-2.0		2
	UV5.33	104.6 +/-6.3**	18.5 +/-1.9		2
	UV5.36	112.1 +/-19.2**	20.4 +/-2.3		2
	UV5.37	NA	NA		
	UV5.38	NA	NA		
	UV5.39	111.2 +/-11.6**	23.6 +/-2.1*		
	UV5.42	107.2 +/-8.5**	22.6 +/-2.9		2
	UV6.3	102.9 +/-5.8	25.8 +/-3.7**	yes	1
	UV6.8	103.6 +/-8.5	28.8 +/-7.2**	yes	1
	UV6.9	100.5 +/-0.3	16.8 +/-1.3*		
	UV6.14	105.6 +/-16.7	21.5 +/-10.4		
	UV6.16	115.3 +/-15.4**	22.0 +/-2.1		2

A first group of 7 mutants (Group 1) developed rhizoids whose diameter was greater than those of wild type and sinuosity was equal or greater than in wild type. The thicker rhizoid phenotype suggests that the domain of cell growth at the apex may be larger in rhizoids of these mutants than in wild type. Furthermore, an additional phenotype was observed in each mutant with thicker rhizoids and greater sinuosity: they developed gaping air pores on the dorsal epidermis in the mature gametophyte; openings were elongated instead of disc-shaped in wild type (S2 Fig). These phenotypic similarities shared by these mutants suggested that each line might harbour mutations in the same gene, i.e. they might be allelic. These mutants were not characterized further in this study because the increase in rhizoid diameter was not observed in wild-type rhizoids treated with microtubule stabilizing or microtubule destabilizing drugs.

A second group of 13 mutants (Group 2) developed rhizoids whose diameter was identical to wild type but whose sinuosity was greater than in wild type. The rhizoid phenotype of this mutant class–the same diameter as wild type rhizoids, but more sinuous–suggests that these mutants polarize growth similarly to wild type but fail to stabilize the growth site to a constant position in the apical dome. We hypothesized that the mutations causing the Group 2 phenotype defined several linkage groups.

### Multiple mutant alleles define Mp*NIMA-RELATED PROTEIN KINASE (*Mp*NEK)* as a regulator of stability of the site of tip growth

**Fig 2:**
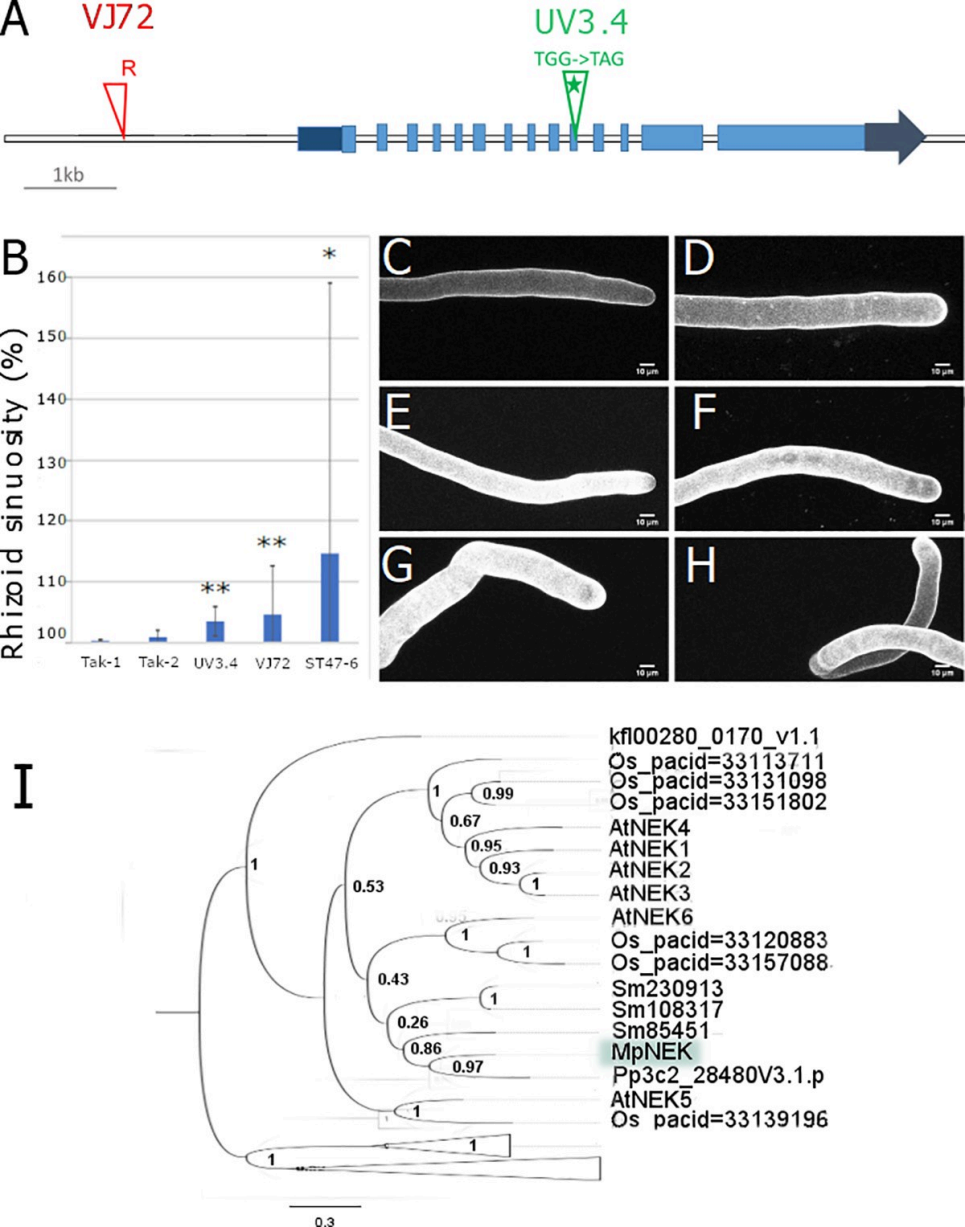
*nek* mutants develop wavy rhizoids. **A:** Schematic representation of Mp*NEK* gene model. UTR regions are represented in dark blue, CDS in light blue. The T-DNA insertion site in VJ72 is indicated with a red triangle. The Right Border position on the T-DNA is indicated with R. The early stop codon is indicated by a green star in UV3.4. **B:** Mean rhizoid 3D sinuosity from 10 mature rhizoids. Error bars indicate +/- SD. Single or double stars indicate a Benjamini-Hochberg adjusted p-value from non-parametric Dunn test lower than 0.05 and 0.01 respectively. **C-H:** Mature rhizoids of Tak-1 (C), Tak-2 (D), UV3.4 (E), VJ72 (F). The weakly wavy phenotype of ST47-6 (G) and strong wavy phenotype of ST47-6 (H). **I**: Phylogenetic tree inferred from full-protein alignment of the NEK orthogroup. The tree is rooted with NEK proteins of chlorophyte algae. Branch support is shown as p-value from SH test. MpNEK is highlighted in a green box. The fully deployed tree can be found in S4 Fig.

To test the hypothesis that Group 2 mutants defined multiple linkage groups, all 14 Group 2 mutants were crossed with each other. We analysed the segregation of rhizoid phenotypes in their progeny. The phenotype of progeny of F1 crosses between T-DNA and UV-B of Group 2 mutant lines–the same diameter as wild type rhizoids, but more sinuous–indicated that 3 of these 14 mutations constitute a single linkage group. Crosses between UV3.4, ST47-6 and VJ72 resulted in 100% mutant progeny (Table 2). The absence of wild type progeny is consistent with the mutations being in the same gene or closely linked on the same chromosome. To identify the causative mutation in UV3.4 we sequenced the genomes of UV3.4 and independent non-allelic UV-B mutants. Then, we compared the mismatches between UV3.4 and the independent non-allelic UV-B mutants. Of the 190,843 mismatches in the initial UV3.4 pool of mismatches, six candidate mutations remained after filtering steps were carried out (Table 3). Of those, only one mutation is a nonsense mutation and it is in the coding sequence of *NIMA-RELATED PROTEIN KINASE* (Mp*NEK)* (Table 3). To verify that the mutation in Mp*NEK* causes the wavy rhizoid phenotype of UV3.4 we tested if there was a mutation in the Mp*NEK* gene of the VJ72 mutant which is in the same linkage group as UV3.4. We first tested if the T-DNA insertion caused the wavy rhizoid phenotype. We scored hygromycin resistance among progeny of the VJ72 line (harbouring a hygromycin resistance gene on its T-DNA) backcrossed to wild type (Table 4). Half of the progeny was hygromycin resistant, consistent with the segregation of a single T-DNA in this population. Of these progeny, all hygromycin resistant plants formed wavy rhizoids, indicating that the T-DNA co-segregated with the wavy mutant phenotype. These data are consistent with the hypothesis that a T-DNA insertion causes the wavy phenotype in mutant plants. To identify the gene mutated by the T-DNA insertion, sequences flanking the T-DNA from VJ72 were isolated by TAIL PCR (S1 Data). The flanking sequences indicated that the T-DNA was inserted in the promoter region of Mp*NEK* 1760 bp 5’ of the predicted start codon (Fig 2). Taken together these data indicate that the nonsense mutation in the in *MpNEK* gene of UV3.4 and a T-DNA insertion in the promoter of Mp*NEK* in VJ72 cause a defective wavy rhizoid phenotype.

**Table 2:** Co-segregation test between UV3.4, ST47-6 and VJ72.

Parent 1	Parent 2	Wavy rhizoid	Straight rhizoid	Total F1	Genetic distance (cM)
UV3.4	ST47-6	156	0	156	0
VJ72	ST47-6	236	0	236	0

**Table 3:** Candidate mutations for UV3.4.

Mutated gene model	Arabidopsis homolog	Annotation of Arabidopsis homolog	Type of mutation
Mp2834s1300	AT3G37750/NEK6	beta-tubulin phosphorylation	nonsense
Mp2145s1300	AT3G54630/EIF4G	Kinetochore protein-like protein	missense
Mp4343s1080	AT2G1677/bZIP23	Zinc deficiency response transcription factor	missense
Mp2528s1180	AT5G16840	Binding partner of ACD11-1, response to cytokinin	missense
Mp1040s1130	AT5G03900	Iron sulphur cluster biosynthesis family protein	missense
Mp4751s1220	No hit	NA	Missense

**Table 4:** Co-segregation analysis of wavy rhizoid and hygromycin resistance phenotypes VJ72xWT F1 population. HygR: hygromycin resistant. HygS: hygromycin sensitive.

Line	Wavy rhizoids		Straight rhizoids		Total F1		HygS/R ratio
	HygR	HygS	HygR	HygS	HygR	HygS	
VJ72	136	0	0	145	136	145	1/0.9

There is a single NEK protein encoded in the *M*. *polymorpha* genome, *P*. *patens*, three in *Selaginella moellendorfii*, six *O*. *sativa* and seven in *A*. *thaliana*. To determine the phylogenetic relationships between these NEK proteins we generated gene trees using maximum likelihood statistics (Fig 2I). MpNEK is a member of a monophyletic group that contains land plant NEK proteins (bootstrap 0.995). Within this group MpNEK is a member of a monophyletic group that includes the single copy *P*. *patens* NEK protein and the three *S*. *moellendorffii* proteins. However, the bootstrap support for this monophyletic group is not strong. The similarity between the morphology of oryzalin and Taxol-treated rhizoids with the *nek* loss of function, is consistent with a role of MpNEK in modulating microtubule dynamics (Figs 1 and 2) required for stabilization of the apex during tip-growth as proposed by [16].

The identification of Mp*NEK* in the screen validates our forward genetic approach to identify proteins that regulate microtubule dynamics and organization during tip-growth. We carried out no further characterization of MpNEK because MpNEK is already known to be involved in tip-growth stability and destabilize microtubules in growing rhizoids of *M*. *polymorpha* (16).

### Multiple mutant alleles define *WAVE DAMPENED-LIKE (WDL)* as a regulator of stability of the site of tip growth

**Fig 3:**
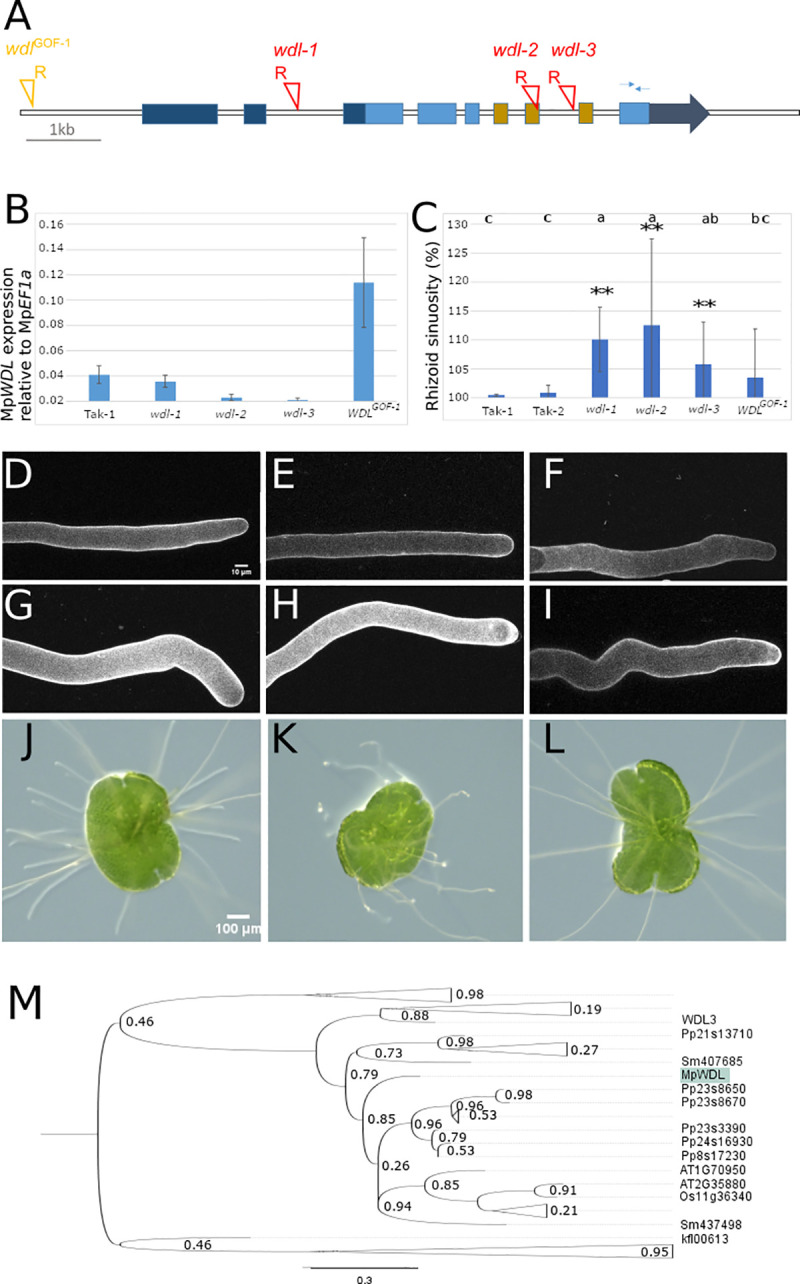
*wdl* gain-of-function and loss-of-function mutants develop wavy rhizoids. **A:** Schematic representation of Mp*WDL* gene model. UTR regions are represented in dark blue, CDS in light blue. The TPX2 domain spans 3 exons represented in brown. T-DNA insertion sites are indicated with red triangles and the location of right border orientation marked with R. The thin arrows on exon 9 represent the positions of the primers used for qPCR. **B:** Steady state level of expression of Mp*WDL* normalized to Mp*EF1a*. **C:** Mean rhizoid 3D sinuosity from 10 mature rhizoids. Error bars indicate +/- SD and two stars indicate a Benjamini-Hochberg adjusted p-value from non-parametric Dunn test lower than 0.01. **D-I:** Mature rhizoids of Tak-1 (D), Tak-2 (E), *wdl*^*GOF-1*^ (F), *wdl-1* (G), *wdl-2* (H) and *wdl-3* (I). **J-L:** 2d old gemma of Tak-1 (J), *wdl-2* (K) and *wdl-2* complemented with *pro*Mp*WDL*::Mp*WDL-YFP*. **M:** Phylogenetic tree inferred from alignment of the TPX2 domain of TPX2 domain-containing proteins. The tree is rooted with TPX2 proteins of chlorophyte algae. Branch support is shown as p-value from SH test. TPX2 proteins that belong to the MpWDL clade. MpWDL is highlighted in green. The fully deployed tree can be found in S5 Fig.

The phenotype of progeny from F1 crosses between T-DNA and UV-B of Group 2 mutant lines–with higher sinuosity than wild type but same diameter as wild type–indicated that 4 of these 14 mutations comprised a second linkage group. CR1, CR2, ST45-1 and ST33-5 T-DNA mutants were crossed to each other. The rhizoids of all (100%) of the F1 progeny were sinuous compared to the straight rhizoid of wild type (Table 5). The absence of wild type progeny is consistent with the mutations being in the same gene or closely linked on the same chromosome. This suggested that each mutant line harbours an independent mutation in the same gene (Table 5). DNA sequences flanking the T-DNAs were identified by TAIL PCR. The flanking sequences demonstrated that the T-DNA of CR1, CR2 and ST45-1 were inserted into a gene encoding a protein that is homologous to the *A*. *thaliana WAVE DAMPENED2 LIKE* [17]. These mutant alleles were designated *wdl-1*, *wdl-2* and *wdl-3* respectively. TAIL PCR failed to amplify sequences flanking the T-DNA in the ST33-5 mutant. To identify the causative mutation in ST33-5, we sequenced the entire genome of ST33-5 and identified a T-DNA insertion site (S1 Data) by aligning sequencing reads against the reference genome and the T-DNA sequence. A T-DNA in ST33-5 was inserted in the promoter region of MpWDL 3436 bp upstream of the start codon. (Fig 3A). The presence of an insertion into the MpWDL promoter region of ST33-5 and the absence of wild type in the next generation when crossed to *wdl-1*, *wdl-2* and *wdl-*3 mutants indicates that the sinuous rhizoid phenotype of ST33-5 results from a mutation in the MpWDL gene.

**Table 5:** Co-segregation test between *wdl-1*, *wdl-2*, *wdl-3* and *wdl-4* mutant lines.

Parent 1	Parent 2	Wavy rhizoid	Straight rhizoid	Total F1	Genetic distance (cM)
ST45-1/ *wdl-1*	ST33-5/ *wdl-4*	237	0	237	0
CR2/*wdl-2*	CR1/wdl-3	188	0	188	0
CR2//*wdl-2*	ST45-1/*wdl-1*	291	0	291	0

T-DNAs are inserted into the 7th exon and 7th intron of the Mp*WDL* gene in *wdl-2* and *wdl-3* respectively (Fig 3A). Insertions in introns and exons often block the production of a stable transcript. To test if *wdl-2* and *wdl-3* produce a functional Mp*WDL* transcript we measured steady state levels of Mp*WDL* mRNA in *wdl-2* and *wdl-3* mutants. MpWDL transcript was not detectable in either *wdl-2* or *wdl-3* (Fig 3B). This suggests that *wdl-2* and *wdl-3* are complete loss of function mutants. Furthermore, the location of the T-DNA insertions in *wdl-2* and *wdl-3* at the predicted microtubule-binding domain TPX2 suggests that even if a truncated protein were produced, it would not be functional. To test the hypothesis that *wdl-2* and *wdl-3* are loss of function alleles, we transformed these mutants with wild type Mp*WDL* genomic sequence that included 5 kb upstream of the transcriptional start site and the coding region (*pro*Mp*WDL*:Mp*WDL-YFP*). The wild type rhizoid phenotype was restored by expressing *pro*Mp*WDL*:Mp*WDL-YFP* in the *wdl-2* and *wdl-3* background (Fig 3J–3L). We conclude that the wavy rhizoid phenotype is caused by the loss of *WDL* function in *wdl-2* and *wdl-3*.

A T-DNA is inserted in the promoter of Mp*WDL* of the ST33-5 *wdl* mutant, with the right border of the T-DNA oriented 5’ to the Mp*WDL* locus. Insertions into the 5’ regulatory regions where the right border of the T-DNA is 5’ to the coding sequence have been reported to be associated with the overexpression of the sequences 3’ of the T-DNA [17]. To test the hypothesis that Mp*WDL* is expressed at higher levels in ST33-5 mutants than in wild type, we measured the steady state levels of *MpWDL* transcript. *MpWDL* transcript levels are 4-times higher in ST33-5 than in Tak-1 wild type (Fig 3B). Furthermore, there are no mutations in the coding sequence of Mp*WDL* in *ST33-5*. These data suggest that the ST33-5 line harbours a gain of function Mp*WDL* mutation in. ST33-5 was named *wdl*^*GOF-1*^.

The fact that both loss of function and gain of function *wdl* mutants develop sinuous rhizoids suggests that loss of MpWDL activity and extra MpWDL activity had a similar effect on the stability of the apex during tip growth. To determine if the loss and gain of function mutants were phenotypically distinguishable, we measured waviness of the rhizoids by calculating their sinuosity. We could not distinguish between the sinuosity of the gain of function mutant *wdl*^*GOF-1*^ and the loss of function mutant *wdl-3* (Fig 3C, 3F and 3I). Because the phenotypes of some *wdl* loss and *wdl* gain of function mutants are indistinguishable–like Taxol- and oryzalin-treated rhizoids at certain concentrations (Fig 1)–these data suggest that the MpWDL protein regulates microtubule dynamics required for the stabilisation of the apex during tip-growth.

### *Mp*WDL-YFP localizes to microtubules and promotes the formation of a longitudinal array of parallel-arranged bundled microtubules in the shank of growing rhizoids

There is a single MpWDL encoding gene in *M*. *polymorpha*, 13 in *P*. *patens*, 6 in *O*. *sativa* and 8 in *A*. *thaliana*. MpWDL proteins are members of the TPX2 domain-containing microtubule binding proteins that contain the KLEEK motif (at position 352 in *Mp*WDL) (Fig 3M). In *A*. *thaliana*, MpWDL proteins promote microtubule growth, and bind to and bundle microtubules *in vitro* [18]. We hypothesize that *Mp*WDL modulates microtubule organization during the growth of the *M*. *polymorpha* rhizoid.

**Fig 4:**
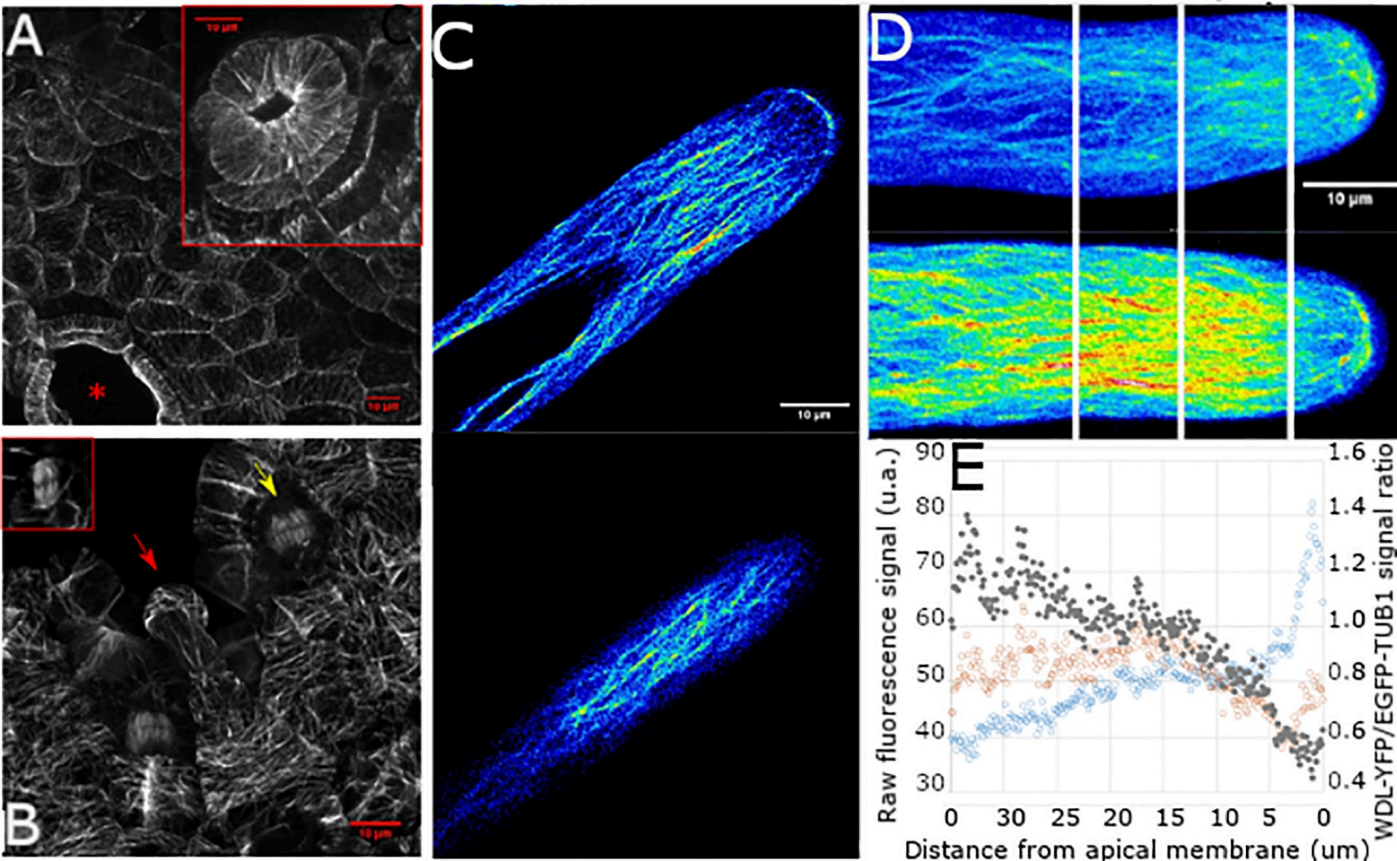
MpWDL-YFP localizes to microtubule bundles of epidermal cells and rhizoids. **A**: Dorsal epidermis of a plant transformed with the *pro*Mp*WDL*:Mp*WDL-YFP* gene construct. The red star indicates a mature air pore and the inserted red box frames a developing air pore. **B:** Meristematic notch of a *pro*Mp*WDL*:Mp*WDL-YFP*. The red arrow points to a mucilage papilla cell directly above the meristematic notch. The yellow arrow points to a phragmoplast, while the red inserted box frames mitotic microtubules during metaphase. **C:** YFP fluorescence in a midplane optical section (top) and cortical plane optical section (bottom) of a growing rhizoid of a plant transformed with *pro*Mp*WDL*:Mp*WDL-YFP*. **D**: GFP and YFP fluorescence in a Z-maximum intensity projection of growing rhizoids of a plant transformed with *pro*Mp*EF1a*:Mp*GFP-*Mp*TUB1* (top) and of a plant transformed with *pro*Mp*WDL*:Mp*WDL-YFP* (bottom). The vertical white lines represent 10, 20 and 30 μm distance from the apex. E: Abundance of MpWDL-YFP signal normalized by GFP-MpTUB1 signal along the longitudinal axis of growing rhizoids (full grey circles, vertical axis on the right hand side). Raw MpWDL-YFP and GFP-MpTUB1 are plotted for reference (orange and blue hollow circles, respectively, vertical axis on the left hand side).

If MpWDL protein regulates microtubules in stabilizing the apex during tip growth, we hypothesized that MpWDL protein would bind to microtubules. To test this hypothesis, we transformed wild type *M*. *polymorpha* with a gene construct in which the *Mp*WDL-YFP protein fusion was placed under the control of the endogenous Mp*WDL* promoter (*pro*Mp*WDL*-Mp*WDL*:*YFP*). We imaged YFP fluorescence in transformed lines. *Mp*WDL-YFP localizes to interphase, spindle and phragmoplast microtubules in all gemma epidermal cells investigated (Fig 4A and 4B). Moreover, the MpWDL-YFP fluorescence distributes homogeneously along the length of microtubules, suggesting that MpWDL does not preferentially decorate the plus-ends of microtubules.

Microtubules are arranged in two distinct domains in the growing rhizoid. Dense bundles of microtubules run longitudinally along the shank of the rhizoid and extend into the apical dome where microtubules are relatively less bundled (S3B–S3E Fig). Microtubules converge on a region just behind the tip of the apex to form a microtubule focus. The distribution of MpWDL-YFP is characteristic microtubule-localized signal and resembles the localisation of GFP-MpTUB1 in growing wild type rhizoids (Fig 4C and 4D). MpWDL-YFP decorates the bundled microtubules along the shank and the microtubule network in the apical dome. This suggests that MpWDL and MpTUB1 co-localize. However, MpWDL-YFP fluorescence is stronger in microtubules along the shank than in the dome (Fig 4D). By contrast, GFP-MpTUB1 fluorescence is stronger in the apical dome and at the position of the microtubule focus than it is along the shank (Fig 4D). Consistent with this observation is the demonstration that the ratio between MpWDL-YFP and GFP-MpTUB1 is higher in the shank of growing rhizoids than in the apical dome (Fig 4E). These data suggest that MpWDL-YFP localizes preferentially to microtubules along the shank in comparison to microtubules in the apical dome. The fact that MpWDL-YFP is more abundant in the region of the cell where microtubules are highly bundled than in regions where microtubules are less bundled suggests that MpWDL may be involved in microtubule bundling.

### MpWDL-YFP promotes formation of a longitudinal array of parallel-arranged bundled microtubules in the shank of growing rhizoids

**Fig 5:**
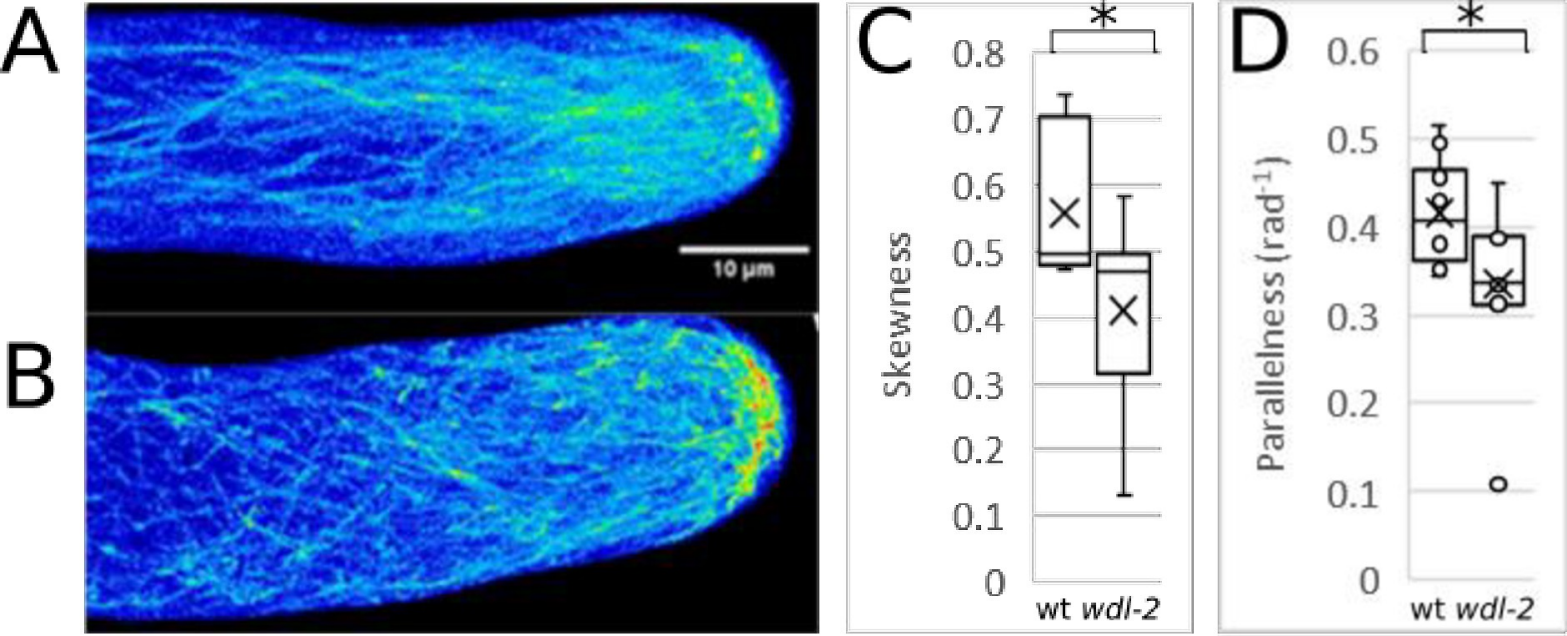
Microtubules are less bundled and parallel in growing *wdl-2* rhizoids. GFP fluorescence in a Z-maximum intensity projection of a growing rhizoid from a plant transformed with *pro*Mp*EF1a*:*GFP-*Mp*TUB1* in wild type (**A**) and *wdl-2* (**B**) growing rhizoids. Colour code corresponds to a 16 colour scale. **C**-**D**: Quantification of the skewness of the signal intensity distribution in pixels corresponding to microtubules (**C**) and microtubule parallelness (**D**) in a cortical section from growing rhizoids in wild type and *wdl-2* mutants (n = 10). Asterisks indicate the p-value from a Mann-Whitney U test lower than 0.05.

Since the MpWDL microtubule-binding protein is more abundant in the rhizoid shank where microtubules are arranged in longitudinal, parallel bundles, we hypothesized that MpWDL was responsible for bundling the shank microtubules. To test this hypothesis, we compared the organization of microtubules decorated with MpGFP-MpTUB1 in growing wild type and *wdl-2* rhizoids (Fig 5A and 5B). Cortical microtubules in the shank of wild type rhizoids are bundled (Fig 5A). Bundles are predominantly parallel. They run longitudinally from base to apex and converge at the tip where microtubule plus ends are located (Figs 5A and S3F and S3G). Cortical microtubules in the shank of growing *wdl-2* rhizoids were significantly less parallel than in wild type and form a “net-like” array (Fig 5B and 5D). Furthermore, cortical microtubules appeared less bundled in *wld-2* rhizoids than wild type. To test the hypothesis that microtubules were less bundled in *wld-2* mutants than wild type, we estimated relative bundling in mutant and wild type rhizoids. The relative bundling of microtubules can be estimated by the skewness in the fluorescence intensity distribution of microtubule pixels (see [19] for an application of skewness as an estimator of actin bundling). The skewness values were significantly lower (Fig 5B and 5C) in the shank of *wdl-2* than in the shank of wild type growing rhizoids (Fig 5C and 5D). This suggests that cortical microtubules in the shank of growing rhizoids in *wdl-2* were less bundled than in the shank growing rhizoids in wild type. These data are consistent with the hypothesis that *Mp*WDL activity is required to bundle microtubules which promotes the formation of the parallel, longitudinal organization of the microtubule cytoskeleton in the shank of growing rhizoids. The wavy morphology and the less parallel, less bundled microtubles in the shank of *wdl-2* loss of function mutants suggest that MpWDL promotes the organization of an array of bundled, parallel and longitudinally orientated microtubules in the shank that participate in stabilisizing the apex where the rhizoid extends by tip-growth.

## Discussion

We demonstrate that proteins that are required for the formation of an array of bundled, parallel and longitudinally orientated microtubules in the non-growing shank stabilize the tip of rhizoid cells during growth. We also confirm that proteins that repress microtubule bundling in the apical dome of rhizoids stabilize tip growth. Taken together, our data indicate that MpWDL and MpNEK proteins act oppositely on microtubule bundling and both are required to stabilize tip-growth. We propose a model where tip-growth stability requires the microtubule bundling-promoting function of MpWDL and the microtubule destabilizing function of MpNEK to be spatially separated, thus maintaining a population of microtubule bundles in the non-growing shank of rhizoids and a population of dynamic microtubules growing toward the apical dome.

We report here that Mp*NEK* is required to maintain the stable position of the growing point in tip-growing cells of *Marchantia polymorpha*. We isolated *nek* mutants from UV-B and T-DNA mutant screens and observed that an early stop codon in Mp*NEK* causes rhizoids to grow wavy instead of straight. Our data are consistent with published data that demonstrate that *nek* loss of function mutant develop wavy rhizoids [16]. Mp*NEK* had not been reported to control tip-growth stability in other land plant models. This may be because there are larger numbers of genes in the *NEK* gene family, with potentially redundant functions in tip growth, in the genomes of other land plant models. This means that loss of function mutations in these genes would not result in defective rhizoid morphology. Because MAP gene families are smaller in *Marchantia polymorpha* than in other land plant models, the probability of observing a defective phenotype is higher in single mutants than in other models like *A*. *thaliana*. Therefore, it is formally possible that identifying the causative mutations in other *Marchantia polymorpha* mutants with wavy rhizoids may reveal new regulators of microtubule-mediated tip-growth stability.

We report that MpWDL is a MAP that regulates tip-growth stability in *M*. *polymorpha*. WDL proteins form a land plant-specific MAP family that promotes microtubule bundling *in vitro* [18]. WDL homologs promote the longitudinal orientation of the cortical microtubule array and repress the anisotropic elongation of root epidermal cells and light-grown epidermal cells in *A*. *thaliana* [18,20,21]. However, they have not been shown to be required for the spatial stability of the site of tip-growth in *A*. *thaliana* although ectopic overexpression results in defective root hair development. In *M*. *polymorpha*, the loss of function *wdl* mutants form wavy rhizoids and microtubules are misaligned in the shank of growing rhizoids: while microtubules are oriented longitudinally in the shank of wild type rhizoids, they are more randomly oriented in *wdl-2*. This suggests that the function of WDL proteins in promoting the longitudinal orientation of microtubules may be conserved in cylinder-shaped cells of land plants that elongate by tip growth.

Taken together, our results suggest that tip-growth stability in *M*. *polymorpha* requires the function of a microtubule destabilizing MAP, MpNEK, and a MAP that promotes microtubule bundling, MpWDL. This is consistent with previous studies in *A*. *thaliana* where both the microtubule destabilizing protein ARK1 [9] and the microtubule bundling protein MOR1 [14] are required to stabilize tip-growth. Microtubules are more bundled in the root hairs of *ark1* mutants in *A*. *thaliana* and the rhizoids of *nek* mutants in *M*. *polymorpha* compared to their respective wild type root hairs and rhizoids [9,16]. Therefore, MpWDL and MpNEK act oppositely on microtubule bundling and both are required to stabilize tip-growth.

We propose that tip-growth stability requires a spatial separation of MAP-mediated microtubule bundling and microtubule destabilization. MAP-mediated microtubule bundling may promote microtubule longitudinal orientation in the shank. In turn, longitudinally oriented microtubules grow toward the apical dome, where plus-end targeted microtubule destabilizing MAPs prevent excessive microtubule stability that would limit the pool of free tubulin available for the polymerisation of a self-renewing population of microtubules that target polarity markers to the apical plasma membrane.

## Material and methods

### Plant growth

Male and female *M*. *polymorpha* accessions Takaragaike-1 (Tak-1) and Takaragaike-2 (Tak-2) respectively provided by K. Ishizaki (Kobe University, Japan) were used as wild-type. T-DNA mutant lines were selected from a T-DNA mutant collection reported in [17]. Plants were grown horizontally and under continuous white light (~60 PPFD) on a Johnson’s medium modified as detailed in [17]. To induce the formation of reproductive organs, one month-old gemmalings were transferred to a 1:3 mixture of medium vermiculite: John Innes No. 2 compost and grown under 150 PPFD white light supplemented with GreenPower LED research modules FR (8727900 809336 00, Phillips).

### Drug treatments

Gemmae were placed on modified Johnson’s medium [17] supplemented with oryzalin, Taxol or DMSO and mature rhizoids were imaged two days later. Oryzalin was dissolved in pure DMSO to produce 0.1 mM and 0.75 mM 1000X stock solutions. Taxol was dissolved in pure DMSO to produce 3.3 mM and 5 mM 1000X stock solutions. Treatment plates were compared to 0.1% DMSO control plates.

### Cloning and plant transformation

Reporter constructs _pro_Mp*EF1*α:*GFP*-MpTUB1 and _pro_Mp*EF1*α:*GFP*-At*EB*1A were kindly provided by Henrik Buschmann (Osnabruck University*)*. Reporter construct _pro_Mp*EF1*α:*YFP*-MpRabA4 was kindly provided by Ian Moore (Oxford University*)*. GW compatible vectors *Mp*GWB207 [22], *HB444 (*pro*Mp*EF1a:*GW*:*term* in pCambia1300 harbouring the *ACETO LACTATE SYNTHASE* chlorsulfuron resistance gene) were kindly provided by Kimitsune Ishizaki (Kobe University).

The open reading frame of Mp*WDL* was amplified from genomic DNA using Phusion High-Fidelity DNA polymerase (NEB) and gene-specific primers (WDL_infusion _F1: ACCAATTCAGTCGACATGAGTGAAGCCGGA and WDL_infusion_GS1_R1: GCCCTTGCTCACCATGGATCCTGATGCAACAGCCA) designed to introduce a 15bp sequence homologous to HB39 and to replace the stop codon by a glycine-serine C-terminal linker. The YFP C-term entry vector HB39 was linearized using the restriction enzymes NcoI and SalI (NEB). The PCR product was gel purified and recombined with double digested HB39 using the In-Fusion kit (#638910, Clonetech). Finally, the Mp*WDL* fusion entry vector was recombined by LR reaction (#12538–200, ThermoFisher) with the destination vector HB444 to produce a constitutive expression construct.

A 5 kb region upstream of the start codon of Mp*WDL* was defined as the promoter region and amplified from genomic DNA using Phusion High-Fidelity DNA polymerase (NEB) and gene-specific primers (pWDL_F1: AAACGACGCCCAGTGCCACCCAATGCTTCAAAGTTTGAAG) designed to introduce a 15 bp sequence homologous to MPGWB207 and a SmaI restriction site downstream of the 5’UTR. Destination vector MPGWB207 was linearized using the restriction enzymes HindIII and SacI (NEB) to remove the _pro_Mp*EF1a* cassette. The promoter amplicon was gel purified and recombined with double digested MPGWB207 using the In-Fusion kit (#638910, Clonetech). The resulting construct was linearized with the restriction enzyme SmaI and ligated with the Gateway Cassette frame A (Invitrogren) to produce a destination vector. The resulting _*pro*_Mp*WDL* destination vector was finally recombined by LR reaction (#12538–200, ThermoFisher) with the Mp*WDL-GS-YFP* entry vector to produce the _pro_Mp*WDL*:Mp*WDL*-*GS*-*YFP* transformation vector.

The expression clones were transformed into *M*. *polymorpha* spores obtained from a cross between Tak-1 and Tak-2 by co-cultivation with *Agrobacterium tumefaciens* GV3101 using a method from [17]. Co-cultivated sporelings were plated on modified Johnson’s medium supplemented with 100 ppm cefotaxime plus 10 ppm hygromycin, 150 ppm gentamycin or 0,15 ppm chlorsulfuron depending on the constructs.

### Reporter lines

Reporter lines for cytoskeleton and endomembrane compartments were generated by transformation of spores obtained from a cross between Tak-1 and Tak-2. Transformants were screened for adequate signal strength and absence of aberrant morphological phenotype. Selected reporter lines were then crossed to mutant backgrounds when required. Because the level of expression of certain reporter constructs increased or decreased after sexual reproduction, the F1 progeny with wavy rhizoids was compared with the F1 progeny with straight rhizoids, rather than with the parental reporter line.

### Confocal imaging

To image growing rhizoids, one-day-old gemmalings were used. To image growth-arrested rhizoids, two-day-old gemmalings were used. Whether rhizoids were growing was tested by imaging rhizoids over the course of several minutes.

Imaging chambers were designed as described in [23], using cavity slides to allow rhizoids to grow without entering in contact with the cover slip. Gemmae were plated on the agar slice before adding perfluorodecalin and transferring to normal growth conditions. Imaging of rhizoids was finally carried out using a 20X or a 60X water immersion lens.

For the purpose of staining with fluorescent dyes, gemmae were grown on Petri dishes with 10 mL of modified Johnson’s medium, rather than in imaging chambers. Gemmalings were immersed in 10 μM propidium iodide for 4 minutes and washed once by removing the staining solution and adding 20 mL ddH_2_O. Imaging was then conducted using a 40X water dipping lens.

All imaging was carried out using a TCS SP5 confocal microscope (Leica). Excitation and emission settings were determined empirically to minimize chloroplast and cell wall autofluorescence bleed-through. The same settings were used for GFP and Venus: excitation with 488 nm and emission between 500 nm and 570 nm. Brightness was adjusted in raw images to reach the point of saturation and the same modifications were applied to all samples to be compared.

### Quantification of microtubule phenotype in growing rhizoids

We imaged 10 growing rhizoids from independent gemmae of either wild type or *wdl-2*_pro_Mp*EF1a*:*GFP-*Mp*TUB1* originating from the same transformation event. The four cortical-most optical slices (395 nm) for 10 rhizoids from independent gemmae were maximum intensity projected and analysed using the LPX package for ImageJ, as described in [19]. A Mann-Whitney U test (or “two sample Wilcoxon” test) was performed to test the similarity between the wild type and *wdl-2* skewness of the distribution of intensity signal in pixels corresponding to microtubules and the wild type and *wdl-2* angles between lines of the skeletonized images.

### Quantification of the ratio between MpWDL-YFP and MpTUB1-GFP signal

Fluorescence was imaged in growing rhizoids of 10 _pro_Mp*EF1a*:GFP-Mp*TUB1* transformed wild type and 13 _pro_Mp*WDL*:Mp*WDL-YFP* transformed wild type. Signal intensity values were extracted along a 35 μm longitudinal line starting from the apical plasma membrane. The ratio between the average signal intensity values for the two reporter proteins was computed at each position to represent the relative MpWDL-YFP abundance.

### Quantification of mature rhizoid morphological phenotype

Rhizoid sinuosity and rhizoid diameter were measured from the apical most region of growth-arrested rhizoids that would fit in a 200 x 100 μm field of view. The quantification of rhizoid diameter was carried out by measuring manually the shortest diameter along the shank of rhizoids every 5 μm. The quantification of rhizoid 3D sinuosity was carried out using a python script. Software to analyse the waviness of rhizoids in 3D was developed in the Python programming language [24] and uses Python Imaging Library [25] (3.3.1), NumPy [26] (1.11.2) and scikit-image [27] (0.12.3) to open and read images. Contours of cells and its inner pixels were segmented in each slice by applying a pixel intensity threshold with OpenCV (2.8.4) [28]. The noise of the image is removed by selecting contours larger than 1% of the total area of the image. The centres of gravity (CoGs) of these contours were calculated and used to sort the contours to represent the rhizoid in 3D. This order, referred to as the path, is determined by defining the shortest possible route between all central points of the contours using a Simulated Annealing algorithm [29] modified to deal with a three dimensional distance calculation for a non-circular path with a fixed starting point. After defining the path, the angle to the x-axis between each CoG and its successor was calculated to define the local direction of the path. The path is split when a significant change in direction occurs. The contours of the resulting fragments of the path are used for a non-parametric regression analysis using Locally Weighted Scatterplot Smoothing (LOWESS) [30], with the Statsmodels [31] (0.6.1) module. Parameterized with frac. set as 0.1. After the regression analysis of all parts, the regressions were assembled to one path. The pipeline from angle calculation to assembly of the regression was performed for both the x-y and x-z axis in order to maintain the 3D dataset. Finally, this assembly is used to calculate sinuosity as defined by the ratio between the total path length after regression by the shortest distance between base and tip of the rhizoid.

### Expression studies

Three-week-old gemmalings were ground in liquid nitrogen using pestle and mortar. cDNA synthesis and qPCR were carried out as described in [32]. Absolute levels of expression were normalized using Mp*EF1a* as a reference gene. Primers used for qPCR were as follows. Mp*EF1a*_forward: CCGAGATCCTGACCAAGG. Mp*EF1a*_reverse: GAGGTGGGTACTCAGCGAAG. Mp*WDL*_forward: GTTGCCTGTCCTCACGATCA. Mp*WDL*_reverse: TCATGACGCTTGGGCAGTAG.

### Genomic DNA extraction

Genomic DNA was isolated from one-month-old plants grown in axenic conditions. Whole tissue was ground in liquid nitrogen using pestle and mortar. Genomic DNA was extracted with 2% CTAB buffer as described in [33]. To assess the suitability downstream applications, the quantity of extracted DNA was measured with a Qubit 2.0 Fluorometer (Invitrogen) and the purity was estimated from absorbance ratios measured with a ND-1000 spectrophotometer (Nanodrop).

### Thermal asymmetric interlaced PCR

Thermal asymmetric interlaced PCR were carried out as detailed in [17].

### UV-B mutagenesis

Fresh sporangia obtained from a cross between Tak-1 and Tak-2 were harvested and surface sterilized with 1% sodium dichloroisocyanurate (Sigma) solution for 4 minutes and subsequently washed three times in ddH_2_0 before plating on square plates with modified Johnson’s medium at a density of 2000 spores per plate. Excess of water on the plates was allowed to dry out in the flow hood before opening the plates in a Benchtop 2UV Transilluminator (UVP) set at 302 nm and equipped with 6 G8T5E UV-B fluorescent lamps (Ushio). Spores were placed facing the UV-B light source with no plastic or medium in between and exposed for a duration of time sufficient to yield 50% kill, which was 60 s in our experimental conditions. Then, irradiated plates were closed and plates kept in the dark at room temperature overnight before being transferred to normal growth conditions. Finally, irradiated sporelings were assessed for survival and screened for mutant phenotypes 14 days after plating.

### Non-allelism-based mutation discovery pipeline

The sequencing of UV-B mutants was carried out with Illumina’s HiSeq-2000 platform by the Oxford Welcome Trust Center for Human Genomics. Raw reads were quality trimmed using Trimmomatic-0.32 [34] and normalized using Khmer-0.7.1 [35] with a k-mer size of 31. Resulting reads were aligned against the genome assembly using bowtie2-2.1.0 set in—very-sensitive-local mode. Alignments were position sorted and mismatches within reads with q quality higher than 35 were extracted using the function sort and mpileup from bio-samtools-2.0.5. Mismatches in regions with coverage exceeding 100X were excluded using the varFilter function from bcftools of the samtools-0.1.9 package. Then, mismatches were retained only if they were supported by more than seven reads and if they appeared sufficiently homozygous based on a negative FQ value or AF1 value higher than 0.5001. Finally, retained mismatches were considered candidate SNPs if they were absent from other sequenced non-allelic lines, Tak-1 and Tak-2, if they were G2A or C2T substitutions or INDELs consistently with the expected UV-B mutation signature, and if they caused a change in the amino acid sequence of a predicted gene.

## Supporting information

S1 Fig **Rhizoid-mediated soil adhesion of wild type (left) and *wdl-3* mutant (right).** Four week-old gametophytes were transferred on soil and grown for 8 weeks before being pulled up by lifting the gametophyte and its rhizoid system in a vertical motion. More soil was attached to wild type than of *wdl-3* suggesting that straight rhizoids anchor the gametophyte to the substratum more effectively than wavy rhizoids.
(DOCX)

S2 Fig Dorsal epidermis phenotype of two-month-old mutants in Group 1 and Tak-1. Disc-shaped air pores in the centre of air chambers on the dorsal side of Tak-1 (**A**). Some or all air pores are either larger or missing in Group 1 mutant ST33-1 (**B**), UV4.31 (**C**), UV4.32 (**D**), UV4.34 (**E**), UV6.3 (**F**), UV6.8. (**G**) and UV5.28 (**H**). The air chambers that form, develop a larger ovoid (more gaping) air pores than wild type. Scale bar represents 10 mm.
(DOCX)

S3 Fig Microtubules and MpRabA4-labeled vesicles in actively growing rhizoids. **A:** Z-maximum projection of *YFP-*Mp*RabA4* fluorescence in a growing rhizoid from a plant transformed with *pro*Mp*EF1a*:*YFP-*Mp*RabA4*. Colour code corresponds to 16 colours scale. **B-D:** Z-maximum projection (**B**), cortical plane (**C**) and midplane (**D**) of a GFP-MpTUB1 fluorescence in growing rhizoids of a plant transformed with *pro*Mp*EF1a*::*GFP-*Mp*TUB1*. Colour code corresponds to 16 colours scale. **E:** Z-maximum projection (left) and midplane (right) of GFP-MpTUB1 fluorescence in the apical dome of a growing rhizoid in a plant transformed with *pro*Mp*EF1a*::*GFP-*Mp*TUB1*. **F-G**: 20 s temporal projection (**F**) and montage (**G**) in the apical dome midplane of GFP-AtEB1 fluorescence of a growing rhizoid from a plant transformed with *pro*Mp*EF1a*::*GFP-*At*EB1* growing rhizoid. In **F**, purple and yellow arrows indicate the start position of two AtEB1 comets; red and green arrows mark the end position of the same two AtEB1 comets. In **G**, arrows follow the trajectory of AtEB1 comets marked in **F**.
(DOCX)

S4 Fig Phylogenetic tree inferred from full-protein alignment of the NEK orthogroup. The tree is rooted with NEK proteins of chlorophyte algae. Branch support is shown as p-value from SH test. MpNEK is highlighted in a green box.
(DOCX)

S5 Fig Phylogenetic tree inferred from alignment of the TPX2 domain of TPX2 domain-containing proteins. The tree is rooted with TPX2 proteins of chlorophyte algae. Branch support is shown as p-value from SH test. TPX2 proteins that belong to the MpWDL clade. MpWDL is highlighted in green.
(DOCX)

S1 Data Sanger sequencing data of TAIL-PCR amplicons. (XLSX)

## References

[1] Jones VAS, Dolan L. The evolution of root hairs and rhizoids. Ann Bot. 2012 Jul;110(2):205–12. doi: 10.1093/aob/mcs136 22730024 PMC3394659

[2] De Baets S, Denbigh TDG, Smyth KM, Eldridge BM, Weldon L, Higgins B, et al. Micro-scale interactions between Arabidopsis root hairs and soil particles influence soil erosion. Commun Biol. 2020 Apr 3;3(1):1–11. doi: 10.1038/s42003-019-0734-6 32246054 PMC7125084

[3] Rounds CM, Bezanilla M. Growth mechanisms in tip-growing plant cells. Annu Rev Plant Biol. 2013;64:243–65. doi: 10.1146/annurev-arplant-050312-120150 23451782

[4] Parton RM, Dyer AF, Read ND, Trewavas AJ. Apical Structure of Actively Growing Fern Rhizoids Examined by DIC and Confocal Microscopy. Ann Bot. 2000;85:233–45.

[5] de Graaf BHJ, Cheung AY, Andreyeva T, Levasseur K, Kieliszewski M, Wu H. Rab11 GTPase-regulated membrane trafficking is crucial for tip-focused pollen tube growth in tobacco. Plant Cell. 2005;17(9):2564–79. doi: 10.1105/tpc.105.033183 16100336 PMC1197435

[6] Bibikova TN, Blancaflor EB, Gilroy S. Microtubules regulate tip growth and orientation in root hairs of Arabidopsis thaliana. Plant J. 1999;17(6):657–65. doi: 10.1046/j.1365-313x.1999.00415.x 10230063

[7] Ambrose C, Wasteneys GO. Microtubule initiation from the nuclear surface controls cortical microtubule growth polarity and orientation in Arabidopsis thaliana. Plant Cell Physiol. 2014;0(0):1–10. doi: 10.1093/pcp/pcu094 25008974 PMC4160572

[8] Anderhag P, Hepler PK, Lazzaro MD. Microtubules and microfilaments are both responsible for pollen tube elongation in the conifer Picea abies (Norway spruce). Protoplasma. 2000;214(3–4):141–57.

[9] Eng RC, Wasteneys GO. The microtubule plus-end tracking protein ARMADILLO-REPEAT KINESIN1 promotes microtubule catastrophe in Arabidopsis. Plant Cell. 2014;26(8):3372–86. doi: 10.1105/tpc.114.126789 25159991 PMC4176440

[10] Hiwatashi Y, Sato Y, Doonan JH. Kinesins have a dual function in organizing microtubules during both tip growth and cytokinesis in Physcomitrella patens. Plant Cell. 2014;26(3):1256–66. doi: 10.1105/tpc.113.121723 24642939 PMC4001382

[11] Schwuchow J, Sack FD, Hartmann E. Microtubule distribution in gravitropic protonemata of the moss Ceratodon. Protoplasma. 1990;159(1):60–9. doi: 10.1007/BF01326635 11537091

[12] Sieberer BJ, Ketelaar T, Esseling JJ, Emons a. MC. Microtubules guide root hair tip growth. New Phytol. 2005;167(3):711–9. doi: 10.1111/j.1469-8137.2005.01506.x 16101908

[13] Timmers ACJ, Vallotton P, Heym C, Menzel D. Microtubule dynamics in root hairs of Medicago truncatula. Eur J Cell Biol. 2007;86(2):69–83. doi: 10.1016/j.ejcb.2006.11.001 17218039

[14] Whittington a T, Vugrek O, Wei KJ, Hasenbein NG, Sugimoto K, Rashbrooke MC, et al. MOR1 is essential for organizing cortical microtubules in plants. Nature. 2001;411(6837):610–3. doi: 10.1038/35079128 11385579

[15] Eng RC, Wasteneys GO. The Microtubule Plus-End Tracking Protein ARMADILLO-REPEAT KINESIN1 Promotes Microtubule Catastrophe in *Arabidopsis*. Plant Cell. 2014 Aug;26(8):3372–86. doi: 10.1105/tpc.114.126789 25159991 PMC4176440

[16] Otani K, Ishizaki K, Nishihama R, Takatani S, Kohchi T, Takahashi T, et al. An evolutionarily conserved NIMA-related kinase directs rhizoid tip growth in the basal land plant Marchantia polymorpha. Development [Internet]. 2018 Mar 1 [cited 2020 Sep 22];145(5). Available from: https://dev.biologists.org/content/145/5/dev154617. doi: 10.1242/dev.154617 29440300

[17] Honkanen S, Jones VAS, Morieri G, Champion C, Hetherington AJ, Kelly S, et al. The mechanism forming the cell surface of tip-growing rooting cells is conserved among land plants. Curr Biol. 2016 Dec;26(23):3238–44. doi: 10.1016/j.cub.2016.09.062 27866889 PMC5154754

[18] Perrin RM, Wang Y, Yuen CYL, Will J, Masson PH. WVD2 is a novel microtubule-associated protein in Arabidopsis thaliana. Plant J Cell Mol Biol. 2007;49(6):961–71. doi: 10.1111/j.1365-313X.2006.03015.x 17319849

[19] Higaki T, Kutsuna N, Sano T, Kondo N, Hasezawa S. Quantification and cluster analysis of actin cytoskeletal structures in plant cells: role of actin bundling in stomatal movement during diurnal cycles in Arabidopsis guard cells. Plant J. 2010;61(1):156–65. doi: 10.1111/j.1365-313x.2009.04032.x 20092030

[20] Liu X, Qin T, Ma Q, Sun J, Liu Z, Yuan M, et al. Light-regulated hypocotyl elongation involves proteasome-dependent degradation of the microtubule regulatory protein MpWDL3 in Arabidopsis. Plant Cell. 2013;25(5):1740–55. doi: 10.1105/tpc.113.112789 23653471 PMC3694703

[21] Sun J, Ma Q, Mao T. Ethylene regulates the Arabidopsis microtubule-associated protein WAVE-DAMPENED2-LIKE5 in etiolated hypocotyl elongation. Plant Physiol. 2015;169(1):325–37. doi: 10.1104/pp.15.00609 26134166 PMC4577400

[22] Ishizaki K, Nishihama R, Ueda M, Inoue K, Ishida S, Nishimura Y, et al. Development of gateway binary vector series with four different selection markers for the liverwort marchantia polymorpha. PLoS ONE. 2015;10(9):1–13. doi: 10.1371/journal.pone.0138876 26406247 PMC4583185

[23] Kirchhelle C, Chow CM, Foucart C, Neto H, Stierhof YD, Kalde M, et al. The specification of geometric edges by a plant Rab GTPase is an essential cell-patterning principle during organogenesis in Arabidopsis. Dev Cell. 2016;36(4):386–400. doi: 10.1016/j.devcel.2016.01.020 26906735 PMC4766369

[24] Rossum G van. Python tutorial, Technical Report CS-R9526. Centrum voor Wiskunde en Informatica (CWI), Amsterdam; 1995.

[25] Lundh F, Ellis M, others. Python imaging library (PIL). Secret Labs AB,<http://www.pythonware.com/products/pil; 2012.

[26] van der Walt S, Colbert SC, Varoquaux G. The NumPy Array: A structure for efficient numerical computation. Comput Sci Eng. 2011 Mar;13(2):22–30.

[27] Van Der Walt S, Schönberger JL, Nunez-Iglesias J, Boulogne F, Warner JD, Yager N, et al. scikit-image: image processing in Python. PeerJ. 2014;2:e453. doi: 10.7717/peerj.453 25024921 PMC4081273

[28] Bradski G. No Title. Dr Dobbs J Softw Tools.

[29] Seshadri A. Simulated annealing for traveling salesman problem. 2006;1–14.

[30] Cleveland WS. Robust locally weighted regression and smoothing scatterplots. J Am Stat Assoc. 1979;74(368):829–36.

[31] Seabold S, Perktold J. Statsmodels: Econometric and statistical modeling with python. In: 9th Python in Science Conference. 2010.

[32] Breuninger H, Thamm A, Streubel S, Sakayama H, Nishiyama T, Dolan L. Diversification of a transcription factor family led to the evolution of antagonistically acting genetic regulators of root hair growth. Curr Biol. 2016 Jun 20;26(12):1622–8. doi: 10.1016/j.cub.2016.04.060 27265398 PMC4920954

[33] Porebski S, Bailey LG, Baum BR. Modification of a CTAB DNA extraction protocol for plants containing high polysaccharide and polyphenol components. Plant Mol Biol Report. 1997 Mar 1;15(1):8–15.

[34] Bolger AM, Lohse M, Usadel B. Trimmomatic: a flexible trimmer for Illumina sequence data. Bioinformatics. 2014 Aug 1;30(15):2114–20. doi: 10.1093/bioinformatics/btu170 24695404 PMC4103590

[35] Crusoe MR, Alameldin HF, Awad S, Boucher E, Caldwell A, Cartwright R, et al. The khmer software package: enabling efficient nucleotide sequence analysis. F1000Research. 2015 Sep 25;4:900. doi: 10.12688/f1000research.6924.1 26535114 PMC4608353

