# Supplementary material for: Microtubule associated protein WAVE DAMPENED2-LIKE (WDL) controls microtubule bundling and the stability of the site of tip-growth in Marchantia polymorpha rhizoids
Source: PLoS Genet. 2021 Jun 4;17(6):e1009533. doi: 10.1371/journal.pgen.1009533 (PMC8177534; doi:10.1371/journal.pgen.1009533)
Supplement: S1 Fig — Rhizoid-mediated soil adhesion of wild type (left) and wdl-3 mutant (right). Four week-old gametophytes were transferred on soil and grown for 8 weeks before being pulled up by lifting the gametophyte and its rhizoid system in a vertical motion. More soil was attached to wild type than of wdl-3 suggesting that straight rhizoids anchor the gametophyte to the substratum more effectively than wavy rhizoids. (DOCX) [file pgen.1009533.s001.docx]

**
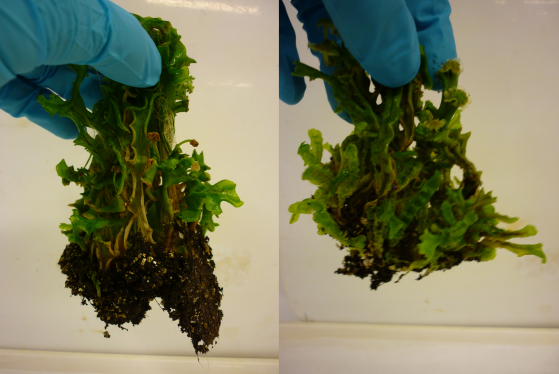
**

**Fig S1: Rhizoid-mediated soil adhesion of wild type (left) and *wdl-3* mutant (right). Four week-old gametophytes were transferred on soil and grown for 8 weeks before being pulled up by lifting the gametophyte and its rhizoid system in a vertical motion. More soil was attached to wild type than of *wdl-3* suggesting that straight rhizoids anchor the gametophyte to the substratum more effectively than wavy rhizoids.**
