# Supplementary material for: Microtubule associated protein WAVE DAMPENED2-LIKE (WDL) controls microtubule bundling and the stability of the site of tip-growth in Marchantia polymorpha rhizoids
Source: PLoS Genet. 2021 Jun 4;17(6):e1009533. doi: 10.1371/journal.pgen.1009533 (PMC8177534; doi:10.1371/journal.pgen.1009533)
Supplement: S2 Fig — Disc-shaped air pores in the centre of air chambers on the dorsal side of Tak-1 (A). Some or all air pores are either larger or missing in Group 1 mutant ST33-1 (B), UV4.31 (C), UV4.32 (D), UV4.34 (E), UV6.3 (F), UV6.8. (G) and UV5.28 (H). The air chambers that form, develop a larger ovoid (more gaping) air pores than wild type. Scale bar represents 10 mm. (DOCX) [file pgen.1009533.s002.docx]

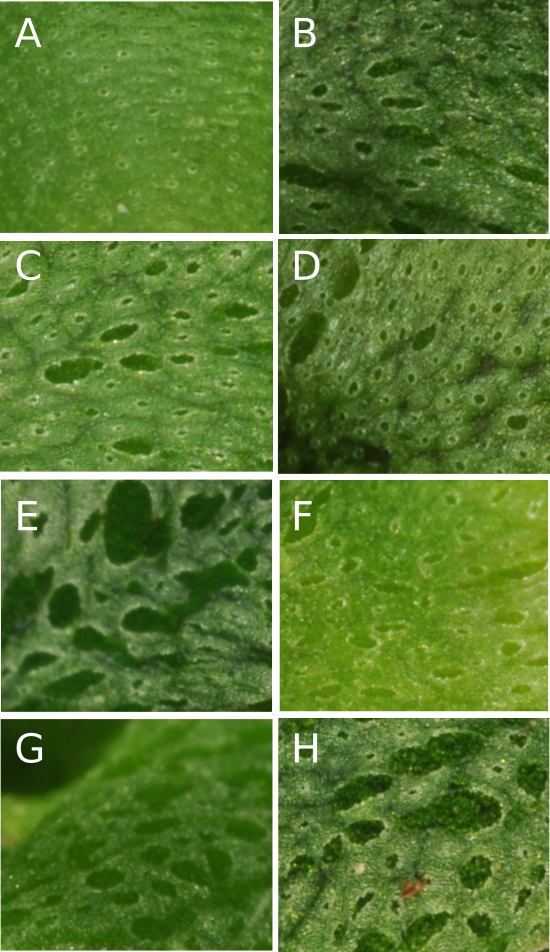


**Fig S2: Dorsal epidermis phenotype of two-month-old mutants in Group 1 and Tak-1.** Disc-shaped air pores in the centre of air chambers on the dorsal side of Tak-1 (**A**). Some or all air pores are either larger or missing in Group 1 mutant ST33-1 (**B**), UV4.31 (**C**), UV4.32 (**D**), UV4.34 (**E**), UV6.3 (**F**), UV6.8. (**G**) and UV5.28 (**H**). The air chambers that form, develop a larger ovoid (more gaping) air pores than wild type. Scale bar represents 10 mm.
