# Supplementary material for: Microtubule associated protein WAVE DAMPENED2-LIKE (WDL) controls microtubule bundling and the stability of the site of tip-growth in Marchantia polymorpha rhizoids
Source: PLoS Genet. 2021 Jun 4;17(6):e1009533. doi: 10.1371/journal.pgen.1009533 (PMC8177534; doi:10.1371/journal.pgen.1009533)
Supplement: S3 Fig — A: Z-maximum projection of YFP-MpRabA4 fluorescence in a growing rhizoid from a plant transformed with proMpEF1a:YFP-MpRabA4. Colour code corresponds to 16 colours scale. B-D: Z-maximum projection (B), cortical plane (C) and midplane (D) of a GFP-MpTUB1 fluorescence in growing rhizoids of a plant transformed with proMpEF1a::GFP-MpTUB1. Colour code corresponds to 16 colours scale. E: Z-maximum projection (left) and midplane (right) of GFP-MpTUB1 fluorescence in the apical dome of a growing rhizoid in a plant transformed with proMpEF1a::GFP-MpTUB1. F-G: 20 s temporal projection (F) and montage (G) in the apical dome midplane of GFP-AtEB1 fluorescence of a growing rhizoid from a plant transformed with proMpEF1a::GFP-AtEB1 growing rhizoid. In F, purple and yellow arrows indicate the start position of two AtEB1 comets; red and green arrows mark the end position of the same two AtEB1 comets. In G, arrows follow the trajectory of AtEB1 comets marked in F. (DOCX) [file pgen.1009533.s003.docx]

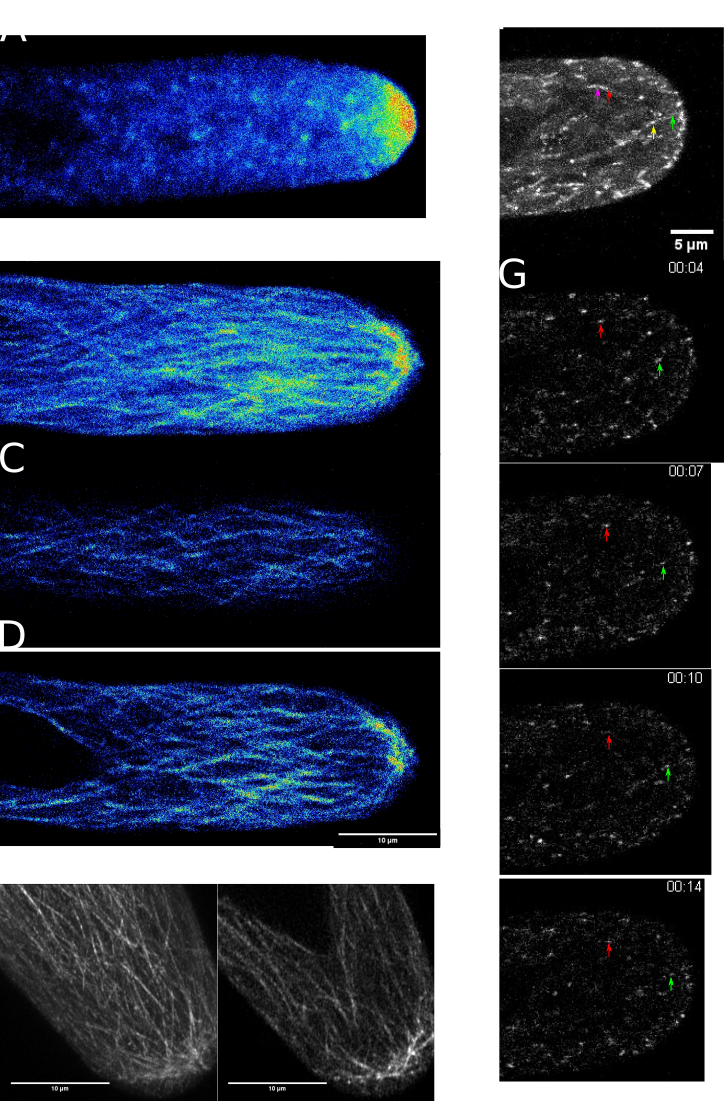


**Fig S3: Microtubules and MpRabA4-labeled vesicles in actively growing rhizoids.** **A:** Z-maximum projection of *YFP-*Mp*RabA4* fluorescence in a growing rhizoid from a plant transformed with *pro*Mp*EF1a:YFP-*Mp*RabA4*. Colour code corresponds to 16 colours scale. **B-D:** Z-maximum projection (**B**), cortical plane (**C**) and midplane (**D**) of a GFP-MpTUB1 fluorescence in growing rhizoids of a plant transformed with *pro*Mp*EF1a::GFP-*Mp*TUB1*. Colour code corresponds to 16 colours scale. **E:** Z-maximum projection (left) and midplane (right) of GFP-MpTUB1 fluorescence in the apical dome of a growing rhizoid in a plant transformed with *pro*Mp*EF1a::GFP-*Mp*TUB1*. **F-G**: 20 s temporal projection (**F**) and montage (**G**) in the apical dome midplane of GFP-AtEB1 fluorescence of a growing rhizoid
