# Supplementary material for: Microtubule associated protein WAVE DAMPENED2-LIKE (WDL) controls microtubule bundling and the stability of the site of tip-growth in Marchantia polymorpha rhizoids
Source: PLoS Genet. 2021 Jun 4;17(6):e1009533. doi: 10.1371/journal.pgen.1009533 (PMC8177534; doi:10.1371/journal.pgen.1009533)
Supplement: S4 Fig — The tree is rooted with NEK proteins of chlorophyte algae. Branch support is shown as p-value from SH test. MpNEK is highlighted in a green box. (DOCX) [file pgen.1009533.s004.docx]

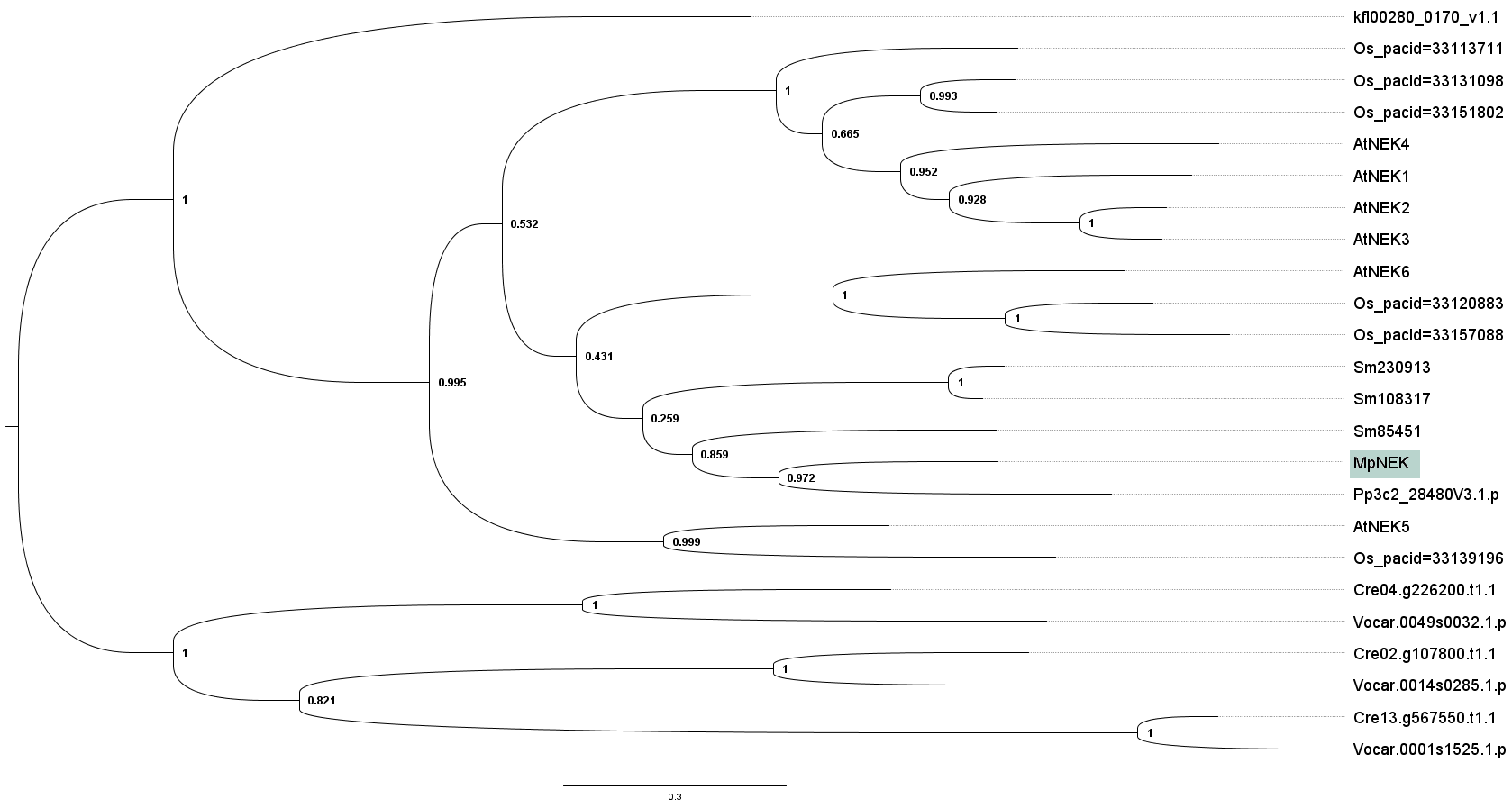


**Fig S4:** Phylogenetic tree inferred from full-protein alignment of the NEK orthogroup**.** The tree is rooted with NEK proteins of chlorophyte algae. Branch support is shown as p-value from SH test. MpNEK is highlighted in a green box.
