# Supplementary material for: Microtubule associated protein WAVE DAMPENED2-LIKE (WDL) controls microtubule bundling and the stability of the site of tip-growth in Marchantia polymorpha rhizoids
Source: PLoS Genet. 2021 Jun 4;17(6):e1009533. doi: 10.1371/journal.pgen.1009533 (PMC8177534; doi:10.1371/journal.pgen.1009533)
Supplement: S5 Fig — The tree is rooted with TPX2 proteins of chlorophyte algae. Branch support is shown as p-value from SH test. TPX2 proteins that belong to the MpWDL clade. MpWDL is highlighted in green. (DOCX) [file pgen.1009533.s005.docx]

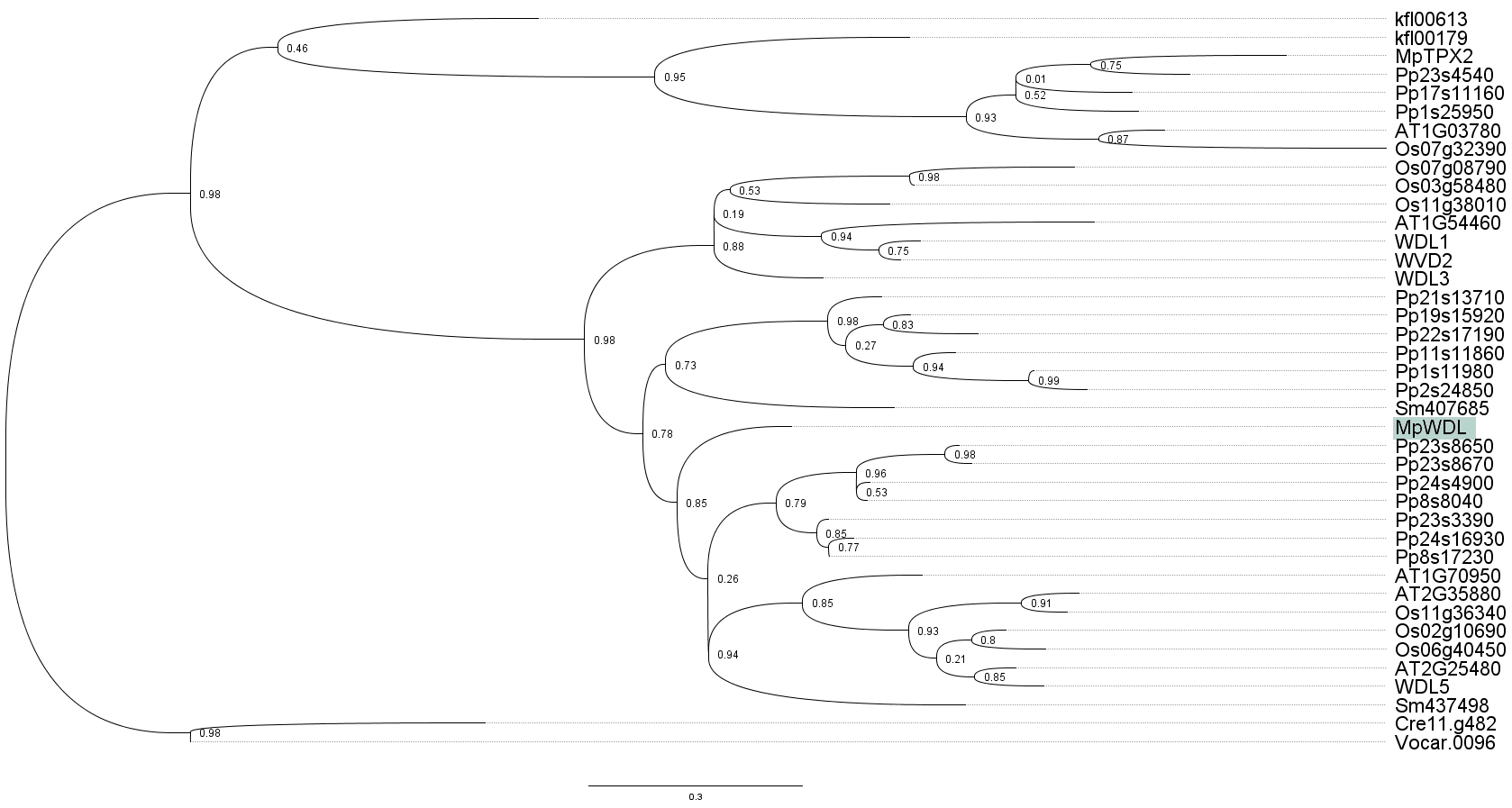


**Fig S5:** Phylogenetic tree inferred from alignment of the TPX2 domain of TPX2 domain-containing proteins. The tree is rooted with TPX2 proteins of chlorophyte algae. Branch support is shown as p-value from SH test. TPX2 proteins that belong to the MpWDL clade. MpWDL is highlighted in green.
